# Genome﻿-wide stress sensitivity moderates the stress-depression relationship in a nationally representative sample of adults

**DOI:** 10.1038/s41598-021-98684-4

**Published:** 2021-10-13

**Authors:** Trent Davidson, David B. Braudt, Robert Keers, Elham Assary, Kathleen Mullan Harris, Jason D. Boardman

**Affiliations:** 1 Department of Sociology, University of Colorado Boulder, Boulder, CO USA; 2 Institute of Behavioral Science, University of Colorado Boulder, Boulder, CO USA; 3 Institute for Behavioral Genetics, University of Colorado Boulder, Boulder, CO USA; 4 Center for Health Outcomes and Population Equity, Huntsman Cancer Institute, Univeristy of Utah, Salt Lake City, UT USA; 5 Department of Biological and Experimental Psychology, School of Biological and Behavioural Sciences, Queen Mary University of London, London, UK; 6 Department of Sociology, University of North Carolina, Chapel Hill, NC USA; 7 Carolina Population Center, University of North Carolina, Chapel Hill, NC USA

**Keywords:** Behavioural genetics, Genomics

## Abstract

We re-evaluate the findings of one of the most cited and disputed papers in gene-environment interaction (GxE) literature. In 2003, a paper was published in *Science* in which the authors demonstrated that the relationship between stress and depression is moderated by a polymorphism in the promoter region (*5-HTTLPR*) of the gene *SLC6A4*. Replication has been weak and led many to challenge the overall significance of GxE research. Here, we utilize data from Add Health, a large, nationally representative, and well-powered longitudinal study to re-examine the genetic determinants of stress sensitivity. We characterize environmental sensitivity using a genome-wide polygenic indicator rather than relying on one polymorphism in a single candidate gene. Our results provide support for the stress-diathesis perspective and validate the scientific contributions of the original paper.

## Introduction

Nearly twenty years ago, Caspi et al.^1^ published a seminal paper in *Science* that set the stage for research in the area of gene-environment interactions (GxE). Their work demonstrated that carriers of the “short” allele in the promoter region (*5-HTTLPR*) of the *SLC6A4* gene were more sensitive to the effects of stress on depression compared to those who were homozygous for the longer repeat allele. To date, replication efforts have been inconsistent^2^ and the authors of a large meta-analysis concluded that there is “no evidence that the serotonin transporter genotype alone or in interaction with stressful life events is associated with an elevated risk of depression in men alone, women alone, or in both sexes combined”^3^. Similarly, researchers have elsewhere detailed the theoretical and statistical shortcomings of GxE research in when it is limited to the candidate gene-environment interaction (cGxE) work^4^. Together, these important criticisms of the cGxE work linking 5-HTTLPR genotype, stress exposure, and mental health may have inadvertently challenged the overall significance of the GxE perspective in general and the genetic origins of environmental sensitivity in particular.

In this paper, we use a large, nationally representative, and contemporary sample to demonstrate the significance of considering the genetic origins of environmental sensitivity as a genome-wide characteristic rather than a single polymorphism in one gene. To illustrate the importance of this perspective we examine a comparable model to that presented in the Caspi et al.^1^ paper but we do not use the cGxE approach. Rather, we use a genome-wide polygenic indicator of overall environmental sensitivity^5^. We also examine the relevance of three similar but distinct GxE models and discuss the importance of considering the phenotype and environmental moderator when examining different models of genetic sensitivity^6^. It is our hope that the results of this paper continue to highlight the far-reaching significance of genetic determinants of environmental sensitivity across the medical, biological, and social sciences.

### Environmental sensitivity—theoretical models

**Figure 1:**
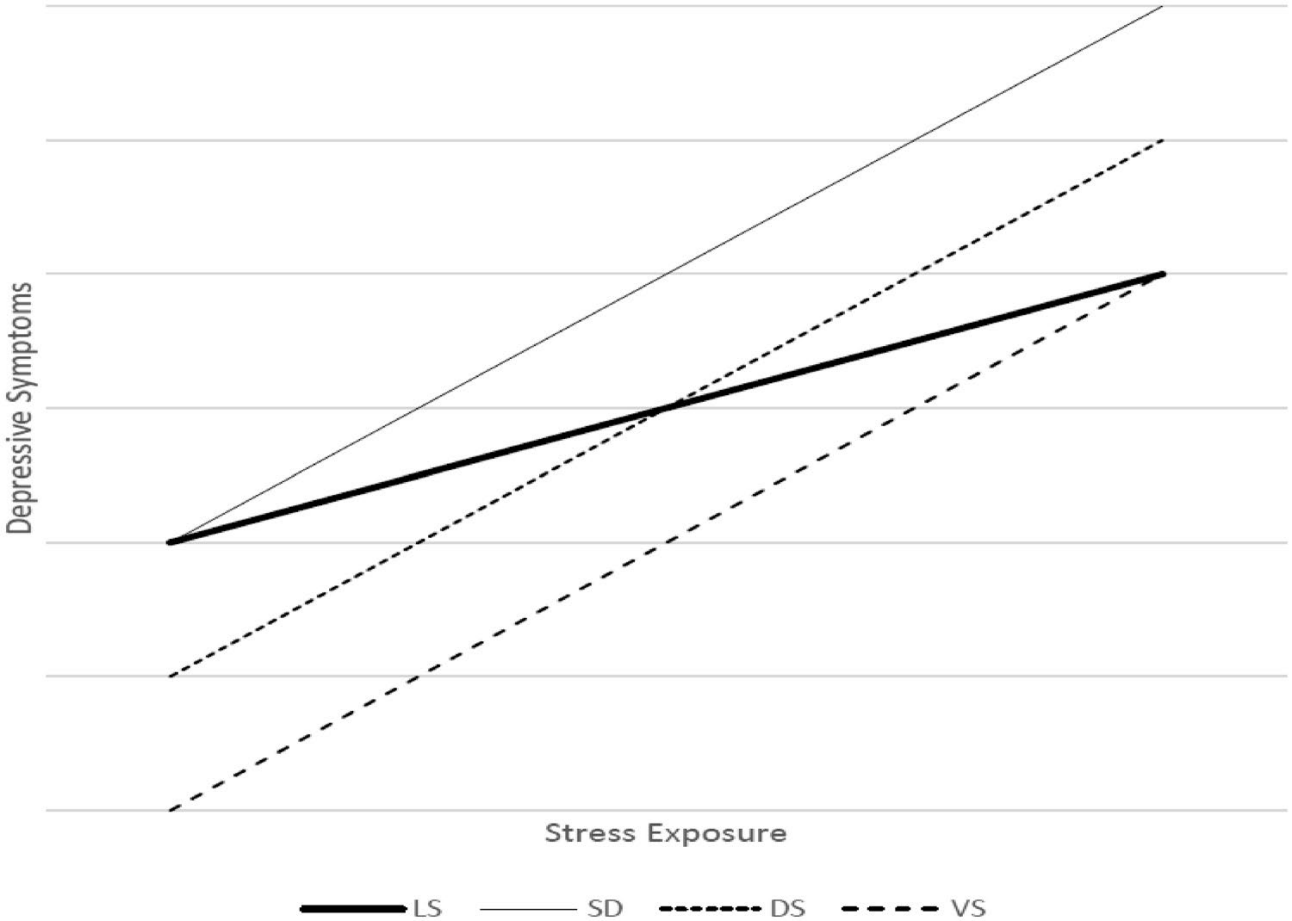
GxE models of depressive symptoms as a function of stress sensitivity.

Figure 1 presents three GxE models related to the notion of environmental sensitivity to stress exposure and subsequent mental health^7^. Each of the three models anticipates that the most sensitive individuals will respond more strongly to stress exposure than the least sensitive (LS), but they differ from one another with respect to the intercept, which has important substantive implications. The results presented by Caspi et al. are best characterized by the stress-diathesis (SD) model. Here, environmentally sensitive individuals and their less sensitive counterparts do not differ from one another with respect to depressive symptoms in the least stressful environments. Differences in overall sensitivity, however, lead to a departure such that the environmentally sensitive individuals report significantly higher levels of depressive symptoms in increasingly stressful environments. The emphasis of this model is on the toxicity of stressful environments rather than the benefits of the least stressful environments, per se.

This distinction is made clearer when one considers the vantage sensitivity model (VS) shown with the bottom, thick-dashed line of Fig. 1^8,9^. As with the SD model, the VS model anticipates that environmentally sensitive individuals may respond more strongly to stress but suggests that these differences will be the most evident in the most positive (rather than low-stress) environments; that is, sensitive individuals derive the greatest psychological benefits in nurturing, supportive, and stress-free environments.

Finally, the Differential Susceptibility (DS) model combines elements of the SD and VS models and suggests that the most environmentally sensitive individuals will report both higher levels of depressive symptoms in the most stressful environments and lower levels of depressive symptoms in the least stressful environments^10^. This relationship is shown by the cross-over line with small dashes in Fig. 1. Thus, all three GxE models will have the same positive interaction term (i.e., the effects of stress on depressive symptoms will be stronger for environmentally sensitive individuals) but the value of the intercept (i.e., the difference in average depressive symptoms in the least stressful environments) differentiates the three models. Examining all three of these models with these updated data is an important contribution to this larger body of work.

The solid bold line (LS) represents the comparison group for all three models; that is, the genotype that is *least sensitive* to the environment. Thus, the other lines represent points at which other genotypes are comparatively *more sensitive* to broad environmental stress than the LS group. The thin, solid line represents the Stress-Diathesis Model (SD*)*, the small-dashed line represents the Differential Susceptibility Model (DS), and the large-dashed line shows the Vantage Sensitivity Model (VS).

### Polygenic sensitivity

The second important contribution of our paper is the application of polygenic score (PGS) techniques to the evaluation of the three models of genetically oriented environmental sensitivity. As described in great detail elsewhere^11^, a PGS is a value that is assigned to each individual that is simply the product of an individual’s genotype at a single nucleotide polymorphism (SNP) and the value of the effect for that loci identified in an independent and well-powered discovery sample, and then summed across the total number of SNPs for which the individual was genotyped. These scores tend to be normally distributed and are standardized to have an intuitive interpretation. An important contribution to work on PGS construction came from Keers and colleagues^5^ who used comparable techniques but instead of focusing on the mean level of an outcome to derive the effect size estimates for each SNP, they focused on discordance among twin pairs to identify the phenotype of environmental sensitivity. Genome-wide regression models were then used to retrieve the beta estimates and risk allele for their overall environmental sensitivity PGS. Thus, reassessing the results of the Caspi et al. paper using an indicator of genetically oriented environmental sensitivity beyond the one candidate gene (i.e., *SLC6A4*) denotes an important contribution to work in this area. To our knowledge, ours is the second paper to apply this PGS to depression longitudinally, but offers a larger and more diverse sample and focuses more broadly on the stress-diathesis relationship^12^.

### Gene-environment correlation and population stratification

Finally, we add to the literature by considering all respondents in the Add Health study for whom genotyped data are available (analytic n = 6472)^13^. Add Health is a nationally-representative, admixed sample of young adults in the U.S., allowing us to expand our analysis beyond individuals of European genetic ancestry, which has unfortunately become the norm^14^. The original paper by Caspi and colleagues only included “Caucasian non-Maori study members” (n = 387) and research since that time, especially work utilizing PGS estimates, has limited the application of summary statistics to individuals from the same genetic ancestral group of the discovery sample. In our analyses, we analyze all genetic ancestry and racial/ethnic groups together for three reasons: (1) theoretically, we do not agree with the belief that the genetic associations for environmental sensitivity differ as a function of one’s racial identity and experience; (2) substantively, the continued stratification of individuals by ethnic classification when examining genetic associations is a problematic practice foreseen nearly 30 years ago in Troy Duster’s *Backdoor to Eugenics* (1990) and the scientific community must work diligently to stop such practices^15^; and (3) methodologically, we are concerned not with a single, causal biological pathway but instead an overall indicator of genetic associations (i.e., a narrow-sense additive genetic variance component). In ancillary analyses we estimate the same models only with those within the European genetic ancestry group and who self-identify as non-Hispanic White to assuage any further concerns; as expected, the results are virtually identical (available upon request). Another possibility is that the sensitivity genotype is correlated with stress exposure (i.e., gene-environment correlation [rGE]). Those who are more sensitive to stressors may make greater efforts to avoid situations in which they may be exposed to additional sources of stress or strain. As others have pointed out^16^, this active form of rGE can make it difficult to interpret the meaning of a GxE interaction term. Accordingly, we estimated a weak baseline correlation between stress and our PGS for environmental sensitivity (r = 0.059, p < 0.001) that loses all significance (r = 0.011, p < 0.490) once controls for genetic ancestry are added.

In summary, in this paper, we reassess the work of Caspi et al. by (1) examining the utility of a genome-wide approach to understanding environmental sensitivity; (2) evaluating our results in terms of an updated theoretical backdrop; and (3) examining similar associations in a different environmental setting (i.e., a different country (U.S.), birth cohort and historical period, among a broader and older age group, and without restrictions to a single race/ethnic group.

## Results

**Figure 2:**
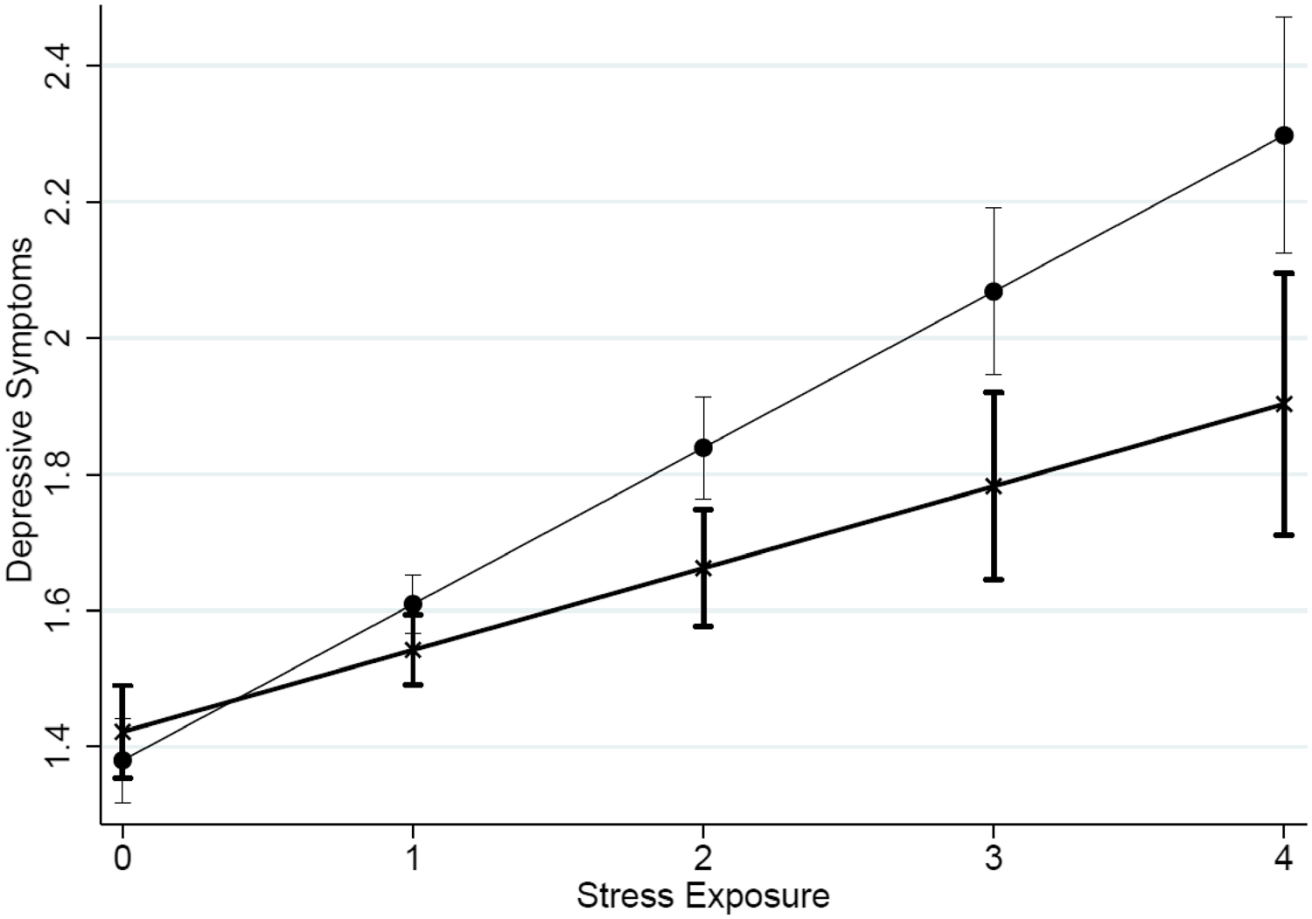
Gene-environment interaction between stress and differential susceptibility genotype as related to depression in adults.

Tables 1 and 2 present the overall descriptive statistics for the analytic sample and bivariate associations between PGS sensitivity and all variables used in the analyses, respectively. Table 3 presents the results from an OLS model in which depressive symptoms are regressed on stress exposure, our environmental sensitivity PGS, and an interaction between the two; Fig. 2 offers a graphical presentation of these estimates. As shown, the models include controls for age, sex, race-ethnicity, educational attainment, and the top five principal components for the full sample of individuals included in the Add Health genetic data^17,18^. The three rows at the top of this table summarize the primary findings of our paper. We report a main effect of stress exposure (b = 0.181, p < 0.000) described in the Methods. Given that the environmental sensitivity PGS is standardized, this estimate reflects the effect of stress on depression for those with an average PGS value. The second value presents the beta estimate for the effect of the PGS on depression. As expected by the stress-diathesis (SD) model, the PGS (b = − 0.009, p < 0.491) is not significantly associated with depression among those with 0 stressful life events. The primary estimates are in bold and provide additional support for the SD model. Specifically, the interaction between stress and the PGS is positive and statistically significant (b = 0.026, p < 0.035). This suggests that the positive association between stress and depressive symptoms is roughly 14.4% stronger among those with a one standard deviation-increase in a genome-wide measure of environmental sensitivity. Figure 2 presents the estimated average value of our depressive symptom measure for individuals with a high (i.e., 75th percentile, line with circles) compared to a low (i.e., 25th percentile, line with x’s) value on the environmental sensitivity PGS. These results support the notion that a genome-wide polygenic measure can capture individual differences in environmental sensitivity. These findings are in line with Caspi and colleagues’ original work and support the SD model emphasizing the noxious nature of stress exposure rather than the salutary nature of a stress-free environment (VS or DS).

**Table 1:** Descriptive statistics for all variables used in the analyses.

	Mean/%	SD/N	Min	Max
Depressive symptoms	1.578	0.590	1.000	4.000
Age (years)	37.959	1.873	33.000	44.167
Sex				
Male	0.428	2771	0.000	1.000
Female	0.572	3701	0.000	1.000
Race-ethnicity				
NH White	0.662	4287	0.000	1.000
NH Black	0.190	1232	0.000	1.000
Native American	0.002	15	0.000	1.000
Asian	0.047	302	0.000	1.000
Hispanic	0.098	636	0.000	1.000
Education				
< High school	0.040	258	0.000	1.000
High school	0.151	979	0.000	1.000
Some college	0.414	2682	0.000	1.000
College degree	0.240	1556	0.000	1.000
Post-baccalaureate	0.154	997	0.000	1.000
Stress exposure	1.015	0.973	0.000	5.000
Environmental sensitivity PGS	0.000	1.000	− 3.518	4.003
Genetic ancestry				
PC1	0.000	1.000	− 0.625	2.584
PC2	0.000	1.000	− 4.611	0.447
PC3	0.000	1.000	− 3.500	7.249
PC4	0.000	1.000	− 13.303	6.762
PC5	0.000	1.000	− 6.849	2.675

All data from Wave V of the National Longitudinal Study of Adolescent to Adult Health (Add Health). N = 6472.

**Table 2:** Bivariate associations between PGS sensitivity and all variables used in the analyses.

	Beta	pr. <
Depressive symptoms	0.044	0.013
Age (years)	0.026	0.133
Sex (male)		
Female	0.026	0.129
Race-ethnicity (NH White)		
NH Black	0.352	0.000
Native American	0.002	0.825
Asian	0.073	0.000
Hispanic	0.051	0.001
Education (< high school)		
High school	− 0.045	0.255
Some college	− 0.070	0.158
College degree	− 0.059	0.172
Post-baccalaureate	− 0.061	0.092
Stress exposure	0.062	0.001
Genetic ancestry		
PC1	0.354	0.000
PC2	− 0.065	0.000
PC3	0.026	0.115
PC4	0.014	0.232
PC5	− 0.047	0.004

All data from wave V of the National Longitudinal Study of Adolescent to Adult Health (add health). N = 6472. All data have been weighted to reflect the sampling design of the Add Health Study.

**Table 3:** The influence of stress on depression as a function of differential susceptibility genotype.

	b	se	t	pr. <	min	max
Age (years)	0.003	0.005	0.640	0.520	− 0.007	0.013
Sex (male)						
Female	0.052	0.020	2.650	0.008	0.013	0.090
Race-ethnicity (NH White)						
NH Black	− 0.152	0.161	− 0.950	0.344	− 0.467	0.163
Native American	0.042	0.221	0.190	0.848	− 0.390	0.475
Asian	0.018	0.138	0.130	0.898	− 0.252	0.288
Hispanic	0.030	0.064	0.460	0.644	− 0.096	0.156
Education (< high school)						
High school	− 0.224	0.062	− 3.610	0.000	− 0.346	− 0.102
Some college	− 0.235	0.058	− 4.010	0.000	− 0.349	− 0.120
College graduate	− 0.275	0.060	− 4.610	0.000	− 0.392	− 0.158
Post baccalaureate	− 0.276	0.062	− 4.450	0.000	− 0.398	− 0.155
**Stress exposure (0–5)**	**0.181**	**0.012**	**15.490**	**0.000**	**0.158**	**0.204**
**Environmental sensitivity PGS**	**− 0.011**	**0.014**	**− 0.780**	**0.438**	**− 0.039**	**0.017**
Stress*PGS	**0.026**	**0.012**	**2.100**	**0.036**	**0.002**	**0.049**
Genetic ancestry						
PC1	0.048	0.063	0.760	0.448	− 0.076	0.172
PC2	0.026	0.027	0.940	0.347	− 0.028	0.079
PC3	− 0.021	0.018	− 1.180	0.238	− 0.055	0.014
PC4	0.003	0.011	0.280	0.779	− 0.019	0.025
PC5	0.000	0.009	− 0.010	0.995	− 0.019	0.018
Intercept	1.506	0.198	7.610	0.000	1.118	1.893

Results of primary interest are boldfaced. Reference category in brackets. Cell entries are as follows: b = unstandardized OLS regression estimates; se = standard error; t = test statistic; pr. ≤  two-tailed p-values; min and max = boundaries of the 95% confidence intervals. All data are weighted to reflect the design of the Add Health Study.

Estimates are derived from Model 3 of Table 3. The thicker line with x’s presents individuals with a low (i.e., 25th percentile) value for the environmental sensitivity PGS. The thinner line with circles shows individuals with a high (i.e., 75th percentile) value for the PGS.

## Discussion

The results presented here are not meant to replicate the results of the Caspi et al. paper directly. Rather, we use this study to demonstrate the continued significance of the GxE framework and to further our understanding of environmental sensitivity, writ large. Importantly, our understanding of environmental sensitivity is an important dimension of research in the social sciences, epidemiology, and public health in which there is already evidence that broad social-environmental factors can limit or enable small genetic associations to become more prominent. As an example, researchers have identified a significant association between stress exposure level and smoking that is moderated by 5-HTTLPR genotype that is nearly identical to the results presented by Caspi et al. but focused on a different outcome. Specifically, among pairs of brothers who are exposed to the same level of stress at the household level, the sibling with more S’ alleles is more likely to smoke in light of increasing numbers of stressors. This same association was not evident among pairs of sisters which is likely due to gender differences in the socialization of appropriate stress-coping behaviors as internalized or externalized^19^. Other work has shown that the relationship between school-level norms regarding cigarette and alcohol consumption and individual-level behaviors is stronger among carriers of the S’-allele in the 5-HTT gene^20,21^. Such “environmentally susceptible” individuals smoke and drink more than they would in other contexts and do so relative to their peers in schools with a high prevalence of these behaviors. These different examples are precisely what Keers and others were trying to capture with their broad indicator of environmental sensitivity linked to genetic loci across the genome^5^. To further illustrate this point we estimate comparable models in which the PGS is calculated for depressive symptoms or major depressive disorder (Tables 4, 5). Both PGSs were positively associated with depressive symptoms but neither significantly interacted with stress to predict depression. While this is an interesting finding that could prove fruitful for future research, the present paper is more broadly focused on global stress sensitivity as a predictor. Taken together with the fact that the PGS estimates for environmental sensitivity are substantively independent from those for major depressive disorder (r = 0.008) and depressive symptoms ( r = − 0.039) (Table 6), these results provide further evidence that this form of environmental sensitivity is unique from genetic pathways affecting depression and depressive symptoms directly.

**Table 4:** The influence of stress on depression as a function of major depressive disorder PGS.

	b	se	t	pr. <	min	max
Age (years)	0.004	0.005	0.7	0.482	− 0.006	0.014
Sex (male)						
Female	0.053	0.020	2.7	0.007	0.014	0.091
Race-ethnicity (NH White)						
NH Black	− 0.147	0.160	− 0.92	0.359	− 0.461	0.167
Native American	0.037	0.228	0.16	0.869	− 0.409	0.484
Asian	0.018	0.136	0.13	0.894	− 0.248	0.284
Hispanic	0.023	0.064	0.35	0.724	− 0.103	0.148
Education (< high school)						
High school	− 0.224	0.062	− 3.62	0.000	− 0.346	− 0.103
Some college	− 0.234	0.058	− 4.01	0.000	− 0.348	− 0.120
College graduate	− 0.272	0.060	− 4.56	0.000	− 0.388	− 0.155
Post baccalaureate	− 0.273	0.062	− 4.4	0.000	− 0.394	− 0.151
Stress exposure (0–5)	0.179	0.012	15.33	0.000	0.156	0.202
PGS MDD	0.020	0.013	1.55	0.12	− 0.005	0.044
Stress*PGS	**− 0.015**	**0.011**	**− 1.31**	**0.189**	**− 0.037**	**0.007**
Genetic ancestry						
PC1	0.055	0.063	0.87	0.382	− 0.068	0.178
PC2	0.027	0.027	0.98	0.325	− 0.026	0.079
PC3	− 0.020	0.018	− 1.11	0.268	− 0.054	0.015
PC4	0.003	0.011	0.25	0.806	-0.019	0.0248
PC5	− 0.000	0.009	− 0.02	0.981	− 0.019	0.018
Intercept	1.496	0.197	7.58	0.000	1.109	1.883

Stress*PGS is boldfaced to highlight. Reference category in brackets. Cell entries are as follows: b = unstandardized OLS regression estimates; se = standard error; t = test statistic; pr. ≤ two-tailed p-values; min and max = boundaries of the 95% confidence intervals. All data are weighted to reflect the design of the Add Health Study.

**Table 5:** The influence of stress on depression as a function of depressive symptoms PGS.

	b	se	t	pr. <	min	max
Age (years)	0.004	0.005	0.730	0.465	− 0.006	0.014
Sex (male)						
Female	0.053	0.020	2.700	0.007	0.015	0.092
Race-ethnicity (NH White)						
NH Black	− 0.142	0.160	− 0.890	0.374	− 0.457	0.172
Native American	0.050	0.222	0.220	0.822	− 0.385	0.485
Asian	0.020	0.136	0.140	0.885	− 0.247	0.287
Hispanic	0.024	0.064	0.370	0.709	− 0.102	0.150
Education (< high school)						
High school	− 0.227	0.063	− 3.610	0.000	− 0.350	− 0.104
Some college	− 0.237	0.059	− 4.000	0.000	− 0.353	− 0.121
College graduate	− 0.275	0.060	− 4.560	0.000	− 0.393	− 0.157
Post baccalaureate	− 0.277	0.063	− 4.410	0.000	− 0.399	− 0.154
Stress (0–5)	0.179	0.012	15.360	0.000	0.156	0.202
PGS depressive symptoms	− 0.009	0.012	− 0.730	0.467	− 0.033	0.015
Stress*PGS	**0.000**	**0.011**	**0.010**	**0.991**	**− 0.022**	**0.022**
Genetic ancestry						
PC1	0.053	0.063	0.840	0.403	− 0.071	0.176
PC2	0.026	0.027	0.970	0.332	− 0.027	0.079
PC3	− 0.020	0.018	− 1.130	0.260	− 0.054	0.015
PC4	0.003	0.011	0.260	0.793	− 0.019	0.025
PC5	0.000	0.009	− 0.040	0.969	− 0.019	0.018
Intercept	1.492	0.198	7.550	0.000	1.104	1.879

Stress*PGS is boldfaced to highlight. Reference category in brackets. Cell entries are as follows: b = unstandardized OLS regression estimates; se = standard error; t = test statistic; pr. ≤  two-tailed p-values; min and max = boundaries of the 95% confidence intervals. All data are weighted to reflect the design of the Add Health Study.

**Table 6:** Correlations between polygenic scores for environmental sensitivity, major depressive disorder, and depressive symptoms.

	PGS environmental sensitivity	PGS MDD	PGS depressive symptoms
PGS environmental sensitivity	1		
PGS MDD	**0.008**	1	
PGS depressive symptoms	− 0.039*	− 0.872***	1

*p < .05, ***p < .001.

## Methods

### Data

National Longitudinal Study of Adolescent to Adult Health (Add Health). Add Health is a nationally representative cohort drawn from a probability sample of 80 U.S. high schools and 52 U.S. middle schools, representative of U.S. schools in 1994–1995 with respect to region, urban setting, school size, school type, and race or ethnic background (n = 20,745, ages 12–20 years at Wave 1 in 1994–1995). Our analyses use data from Wave V which was conducted during 2016–2018 to collect social, environmental, behavioral, and biological data with which to track the emergence of chronic disease as the cohort advanced through their fourth decade of life. Importantly, the Wave V survey was expanded to obtain retrospective reports of birth and childhood circumstances to supplement existing early life data.

Wave V contains a total of 12,300 respondents of which 7033 had genome-wide data. After removing those with missing information on depressive symptom, our final sample contained a total of 6472 respondents. Descriptive statistics for this sample are shown in Table 1.

At Wave IV, Add Health collected Oragene saliva samples from consenting participants (96% of n = 15,701), and requested a second consent to archive their samples for future genomic studies. Approximately 80% consented to archive and were thus eligible for genome-wide genotyping^2^. Genotyping was completed over three years funded by R01 HD073342 (PI Harris) and R01 HD060726 (PIs Harris, Boardman, and McQueen). Add Health utilized two Illumina platforms for genotyping: the Illumina Human Omni1-Quad BeadChip for the majority of samples and the Illumina Human Omni-2.5 Quad BeadChip for the remainder. The two platforms utilized tag SNP technology to identify and include over 1.1 million and 2.5 million genetic markers, respectively, from Omni1 and Omni2.5 derived from the International HapMap Project and the most informative markers from the 1000 Genomes Project (1KGP). The genetic markers include known disease-associated SNPs from multiple sources, ancestry-informative markers, sex chromosomes, and ABO blood typing markers. The platforms also included probes for the detection of copy number variation (CNV) covering all common CNV regions and more than 5000 rare CNV regions. After quality control procedures, genotype data were available for 9974 individuals: n = 7917 from the Illumina HumanOmni1-Quad chip and for 2057 individuals from the Illumina HumanOmni2.5-Quad chip. After filtering, the Add Health genotype data contained n = 609,130 single-nucleotide polymorphisms (SNPs) common to both chips.

### Measures

Our primary outcome of interest, depression, is a concatenation of several questions asked in the interview. Specifically, we create a four-point scale measuring how frequently the respondent reported (1) being unhappy, (2) unable to “shake the blues,” (3) felt sad, or (4) felt depressed (self-diagnosed). Our scale is coded such that 1 = Generally Happy/Good Mood, while 4 = Extremely Unhappy across the aforementioned variables. Our measure of environmental stress was designed to capture the components/dimensions of stressed referenced in the original paper by Caspi et al.^1^. Specifically, we incorporated questions from Wave V concerning employment/job stress, financial stress, housing stress, physical/mental health stress, and relationship stress into an overall, five-point summative measure, with a value of 1 representing generally low stress and 5 representing generally high stress. Our measure of genetic susceptibility to stress is captured by a PGS based on summary statistics from Keers et al.^5^, who instead of focusing on the mean level of an outcome to derive the effect size estimates for each polymorphism, emphasized discordance among twin pairs to identify the phenotype of environmental sensitivity. Genome-wide regression models were then used to retrieve the beta estimates and risk allele for their overall environmental sensitivity PGS. Our models also control for the first five genetic principal components, as well as age, biological sex, race/ethnicity, and educational attainment.

PGSs are calculated as a weighted sum, such that the raw PGSs for environmental sensitivity are calculated as:

$$PGS_{ESi} = \mathop \sum \limits_{j = 1}^{k} \beta_{j} SNP_{ij}$$

where *SNP*_*ij*_ is the allele frequency of the *j*th SNP for the *i*th individual and β_*j*_ is the estimated association between *SNP*_*j*_ and within-pair variability in emotional problems among monozygotic twins as reported by Keers et al.^5^. The raw PGSs are then standardized (μ = 0 and σ = 1) within ancestry groups to account for between-group population stratification.

The Add Health genotyped sample is restricted to four genetic ancestry groups: (1) European, (2) African, (3) Latin American, and (4) East Asian. To identify respondents in these four genetic ancestry groups, a principal component analysis is conducted on all unrelated members of the full genotyped sample. Estimates are then projected onto the remaining related individuals. Each genetic ancestry group is defined by distance from the mean of the first two principal components of the genetic data. To be included in the Latin American, East Asian, and European ancestry groups individuals must be within ± 1 standard deviation of the mean of the first two principal components of the genetic data estimated from all individuals in the Add Health genome-wide data who self-identified as Hispanic, Asian, and non-Hispanic White, respectively. To be included in the African ancestry group individuals must be within ± 2 standard deviations of the mean of the first principal component and ± 1 standard deviation of the mean of the second principal component estimated from all individuals in the genome-wide data who self-identified as non-Hispanic Black.

While genetic ancestry and race/ethnicity are correlated (r = 0.89), they are distinct constructs and attempts to conflate the two are problematic. More specifically, population stratification refers to differences in genetic variation between geographical ancestry groups. Due primarily to the genetic bottle neck created by the small number of humans (~ 2000) who migrated out of Africa early in human history and the tendency for people to procreate with individuals from the same or nearby geographic regions, genetic variance across the entire genome is highly correlated with geography (see^22^ for more detail). However, genetic ancestry should not be conflated with race or ethnicity. Race and ethnicity are social constructs based on a multitude of factors, of which genetic ancestry may or may not be included depending on historical and societal differences in racialization^23^. Consequently, not all individuals included in a given genetic ancestry group may self-identify or be classified by others as the same race and/or ethnicity as other members of their genetic ancestry group.

See^24^ for more details on the Add Health GWAS sample.

### Statistical analyses

Models were estimated using OLS regression with the appropriate sampling weights to reflect the study design of Add Health. Our Stata .do-file (i.e., syntax script) with full coding of variables and models is available upon request.

## Data Availability

All data are publicly available. See https://addhealth.cpc.unc.edu/ for detailed information to access the phenotype and genotype data for the Add Health Study. All analyses were completed using Stata 16 (StataCorp. 2019. *Stata Statistical Software: Release 16*. College Station, TX: StataCorp LLC.) Stata .do-files used for the analyses are available upon request.
